# Knowledge of patients with sarcoma about their illness—a cross-sectional questionnaire-based study (KNOWSARC)

**DOI:** 10.3332/ecancer.2022.1467

**Published:** 2022-11-03

**Authors:** Bharath BG, Vishwas Kumar Anand, Simran Kaur, Nishkarsh Gupta, Sameer Rastogi

**Affiliations:** 1 Department of Medical Oncology, All India Institute of Medical Sciences, New Delhi 110029, India; 2 MBBS, All India Institute of Medical Sciences, New Delhi 110029, India; 3 Department of Physiology, All India Institute of Medical Sciences, New Delhi 110029, India; 4 Department of Onco-Anaesthesia, All India Institute of Medical Sciences, New Delhi 110029, India

**Keywords:** sarcoma, support group, knowledge of sarcoma, India, rare cancer

## Abstract

**Objective:**

To explore the knowledge of Indian patients with sarcoma about their illness in the sarcoma medical oncology clinic of a tertiary care centre.

**Method:**

This prospective cross-sectional questionnaire-based study was done on patients attending the adult sarcoma clinic at the All India Institute of Medical Sciences, New Delhi. Patients aged between 18 and 60 years who gave consent and could understand Hindi or English were recruited for the study. A questionnaire (bilingual – English/Hindi) was given to the patients in the language they understood. The questionnaire captured the knowledge of patients about their illness (cancer/sarcoma), sub-type of sarcoma, the occurrence of the disease (common or rare), origin (bone or soft tissue), metastatic or non-metastatic and the understanding of the possibility of recurrence/progression.

**Result:**

There were 102 patients in the study with a median age of 31.5 years. About 62% of patients had soft tissue sarcomas, and the rest had bone sarcomas. The most common sarcoma in the studied population was Ewing’s sarcoma (23.5%). Metastatic disease was present in 48 (47.1%) of the total patients studied. About 87.2% of patients were aware that they had some form of cancer, and only 62 (69.9%) patients said that they had sarcoma. Only 55 of the 102 patients (56%) knew that the illness was rare. About 70.6% of patients knew about their disease’s actual stage. More patients with metastatic disease understood the stage correctly (35 of 54 patients) as compared to patients with the non-metastatic disease (37 of 48 patients) (77% versus 64.8%, *p* = 0.001). About 77% of patients reported the site of origin of cancer correctly. The patients who had a higher level of education and belonged to a higher socioeconomic status had significantly better knowledge regarding the diagnosis, stage, rarity and prognosis of the disease.

**Conclusion:**

Our patients have poor knowledge about different types of sarcomas, and very few patients know that sarcoma is a rare malignancy. The most crucial factor that influenced the knowledge was the level of education. Through this study, we could identify the sub-group wherein the knowledge gap was significant. Thus, active patient education programmes can help these patients to identify their illness and henceforth therapeutically manage it more wisely.

## Introduction

The incidence of cancer has been on an increasing trend globally. About 70% of the deaths from cancer occur in low- and middle-income countries [1]. India is classified as a low- and middle-income country according to the World Bank country classification. There were 1.3 million new cases of cancer diagnosed and 0.85 million deaths due to cancer in 2020. The diagnosis and prognosis of cancer is a complex and multistep process. One of the many factors is the active communication between the physician and the patient about understanding the disease, diagnostic process, therapeutic options and perception of curability [2]. Another important factor that determines the clinical outcomes of the patients is health literacy.

A recent study done by Gupta *et al* [3] evaluated the impact of health literacy and cognition on severe adverse events in patients with cancer on chemotherapy. This study reported 47.8% of patients had inadequate health literacy, 27.2% had marginal health literacy and 25% had adequate HL. Furthermore, they elaborated those with low levels of health literacy and cognitive abilities have severe levels of adverse drug reactions related to chemotherapy [3]. Similarly, another US-based study demonstrated the prevalence of low health literacy (14%) in surgical oncology patients and its association with increased post-acute care needs and adverse clinical outcomes [4].

The literature suggests that a better understanding of the disease helps patients to make informed choices regarding their treatment decisions [5]. Patients tend to form cognitive representations of the illness based on available information and previous experiences [6]. Hence, better knowledge could potentially improve their coping strategies and quality of life or, on the other hand, prepare them for end-of-life care [7].

A questionnaire-based prospective study by Santoso *et al* [8] evaluated the knowledge of American patients about their understanding of cancer (involved any type of cancer enrolled during the study) (diagnosis, location, stage, status) as a function of age, race, education, income and marital status. Most patients who participated in that study were aware of their diagnosis, but only 23% answered correctly about their cancer stage. The younger population had better knowledge of the cancer stage. Other factors associated with better knowledge include higher income and female gender [8]. Similarly, in a study conducted by Sivendran *et al* [9] which included American patients, about 50% of patients reported their stage correctly. In this study, patients with better knowledge were female, younger than age 65 years, having higher education and higher income. In the same study, understanding of patients about their cancer during or post-treatment was assessed and only about 64% of patients knew exactly their cancer status (i.e. free of cancer or active disease) [9]. Available studies in the literature are contradictory about the patient’s understanding of the clinical course of their illness when they have early-stage versus late-stage [9–11]. Previous studies have even shown that only about half (57%) of cancer patients knew their stage at diagnosis [12]. Lack of understanding about one’s own stage and prognosis can affect their decision and discussion with the treating doctor regarding the approach towards disease management. Overestimation of the stage when the patient is curative or non-metastatic might lead to anxiety, fear and wrong treatment choices. Similarly, overly optimistic views might lead to more use of intensive treatment towards the end of life leading to inappropriate choices.

The majority of previous studies have been reported on common cancers like breast, lung, etc. But there are hardly any studies that assessed knowledge of sarcoma patients regarding their illness. Sarcoma is a relatively rare cancer and it is pertinent that patients know about the rarity of sarcomas, since it can often lead to delay in diagnosis or even misdiagnosis. This may necessitate a second opinion from the expert centres for the management plan. Treatment delay, referral pattern, choice of treatment and compliance with treatment all affect survival outcome in sarcoma patients. Due to the early age of presentation, the years of life lost can be substantial in these patients [13]. The patient’s active involvement in the course of treatment can help to understand the importance and preferences in making decisions that are apt for them [14]. Thus, it becomes all the more relevant to study the perception and knowledge about sarcoma in the young population, wherein timely intervention can help improve the patient’s prognosis. Along with that, it would be interesting to know patients’ perspectives about the cause of sarcoma.

Our hospital has a dedicated sarcoma clinic and is a referral centre for sarcoma across India. Additionally, we have a support group (Sachin Sarcoma Society) that holds weekly support group meetings and imparts knowledge and education for patients with sarcoma. The doctors from our team actively participate in the sessions in form of lectures, presentations and active interactions (twitter handle @Sachinsarcoma). All patients are registered for the support group on a voluntary basis.

Keeping this in mind, the current study was designed to know the patient’s perspective of the disease and focus on the subgroup which needs active counselling. It might be helpful insights for sarcoma physicians to improve their patient understanding and thus better mutual decision-making.

## Material and methods

### Study design

It was a prospective cross-sectional questionnaire-based study of all patients who came to the outpatient department in the sarcoma medical oncology clinic at the All India Institute of Medical Sciences, New Delhi, from 1 May 2019 to 15 June 2019. Patients were recruited according to the following inclusion criteria, i.e., the patients diagnosed with sarcoma, who gave consent to participate, aged between 18 and 60 years and able to understand Hindi or English. The patients who didn’t give consent or had a past or present history of neuropsychiatric or neurodegenerative disorder were excluded. The ethical clearance was taken from the Institute’s ethics committee (IECPG-192/27.03.2019).

### Study protocol

As a protocol, all the consecutive patients who visited the OPD for the first time were counselled about the disease and outcomes with therapy. All patients underwent educational session with the sarcoma support group (Sachin Sarcoma Society). As per the above-mentioned inclusion and exclusion criteria, patients were recruited for the study. The demographic and socioeconomic details (Table 3) were recorded. We used a modified Kuppuswamy’s scale to ascertain the socio-economic status of the participants [17]. After taking the consent from the patients, a questionnaire (bilingual (English/Hindi)) was self-administered by the patients in the language they understood at the time of initial evaluation (Annexure 1). It consisted of core questions related to knowledge of their illness (cancer, sarcoma), sub-type of sarcoma, the occurrence of the disease (common or rare), origin (bone or soft tissue), metastatic or benign and the possibility of recurrence/progression. The constructs of the questionnaire were identified, and items were created in simple format with language familiar to the respondents [18]. The face validation of the questionnaire was done in both English and Hindi languages by six experts (two from medical oncology, one from physiology, one faculty from anaesthesia, one undergraduate student and one patient advocate). Since the questionnaire was used to assess the knowledge of sarcoma patients, therefore the experts decided to keep it as a self-administered form. The experts subjectively judged the operation of a construct used in the questionnaire with respect to how easily the items/questions in the questionnaire were understood, including its feasibility, style formatting, readability, clarity in language and unambiguous understanding and response [19]. The problem was defined, and experts chose it as closed-ended questions, which were quantitatively analysed. We agreed to use multiple-choice type questions with pre-defined options. The questions were administered mindfully, not being the leading question. The sequence of the question was agreed upon by all experts. No pilot studies were done for the current study. The patient advocate made sure that the questions were well-readable and validated to be used in the given patient population.

Concordance between patient information and actual data was confirmed using available case records. Selected patients’ characteristics (age, gender, years of schooling and socioeconomic status) were compared between those who were discordant and those who were concordant for each of the variables tested.

### Statistical analysis

Data were recorded in a predesigned proforma. Continuous variables were summarised as mean and standard deviation (SD) or median and range (for non-parametric data). Quantitative variables were analysed using parametric (Student’s *t*-test) or nonparametric tests (Kruskal–Wallis test), as applicable. All qualitative variables were summarised as frequency (percentage) and were analysed with Chi-square or Fisher’s exact test. The statistical analysis was performed using SPSS version 24 software. A *p*-value of less than 0.05 (<0.05) was considered statistically significant.

## Results

A total of 102 patients diagnosed with sarcoma and who visited our clinic for the first time during the study period were evaluated. The median time from diagnosis of patients with the disease to conduct of this study was 1 year (range: 1 month–6 years).

The median age of the patients was 31.5 (SD +/− 14.37) years (age range, 18–75 years). The majority of patients were males (*n* = 64, 62.7%). The rest of them were females (*n* = 38, 37.3%). A total of 96 (94.1%) out of 102 patients were educated and the remaining 6 were illiterate. The education status was divided into two levels: graduates and non-graduates. Out of 96 patients who were educated, 48 (50%) were graduates and the other 48 (50%) were non-graduates.

A total of 53 (51.95%) patients belonged to lower socioeconomic status as per Kuppuswamy’s classification and the remaining 49 (48.05%) patients belonged to either upper or middle class as described in Table 3.

Amongst the 102 patients, 64.7% (*n* = 66) patients had soft tissue sarcomas and 35.3% (*n* = 36) had bone sarcomas. The most common sarcoma was Ewing’s sarcoma (*n* = 24, 23.5%) followed by synovial sarcoma (*n* = 18, 17.6%). The rest of the sarcoma types are listed in Table 1.

At the time of the questionnaire, 48 (47.1%) of the total studied population had metastatic disease and the remaining 54 (52.9%) patients had a non-metastatic disease.

We included six variables to assess the knowledge of the patients. These include, whether the diagnosis was cancer or not, sarcoma or not, type of sarcoma (bone or soft tissue sarcoma), site of sarcoma, stage of sarcoma (metastatic or no-metastatic) and rarity (is sarcoma a rare disease or common disease). We divided patients according to gender (male versus female), age (less than or equal versus more than 31 years), education (less than or equal to 12 years versus more than 12 years of schooling), level of education (graduate versus non-graduate) and socioeconomic status (lower class versus middle and upper class).

It was found that 87.2% (*n* = 89) of patients were aware that they had some form of cancer. The remaining 11 patients told us that they don’t know whether the diagnosis is cancer or not and 2 patients said they don’t have cancer. The factors that made a difference in the knowledge of cancer were education and socioeconomic status. A higher proportion of educated people said that they have cancer than non-educated persons.

Out of the total 89 patients who said that they have cancer, 62 (69.9%) patients said that their cancer was sarcoma. More patients with a higher level of education and belonged to higher socioeconomic status did statistically better compared to their respective counterparts when it comes to knowledge of cancer and sarcoma diagnosis. The rest of the parameters like gender and age difference had no impact on this knowledge.

Only 53.9% (*n* = 55) of patients knew that their illness is a rare disease, 32.3% (*n* = 36) did not know about the rarity and 10.7% (*n* = 11) reported that it is a common illness. The level of education and socioeconomic status were the statistically significant factors that played a role in knowing the rarity of sarcoma. None of the other evaluated factors showed a difference in patients knowing whether they have rare or common cancer.

Further , we asked patients who knew they had sarcoma their actual diagnosis. About 48 of the total 62 patients knew their disease name. All these 48 patients were literate. Those who reported the name of the sarcoma (*n* = 48) had 100% concordance when cross-checked with hospital records.

In the next question, we asked the patient, what was the tissue of origin of cancer (bone or soft tissue). Of the total 102 patients, 77 patients knew the type of sarcoma correctly and 9 patients answered wrongly. The remaining 13 patients didn’t know the type of sarcoma they had. None of the factors made a significant difference in knowing the type of sarcoma. When asked about risk factors, 84.2% (*n* = 85) patients said they do not know, others said lifestyle (*n* = 5) followed by trauma (*n* = 4), pollution, genetics and smoking (*n* = 2 each) and age (*n* = 1) as risk factors of their disease.

It was seen that 70.6% (*n* = 72) of patients correctly reported the stage of their disease, i.e., either metastatic or non-metastatic. About 21.5% (*n* = 22) patients didn’t know about the metastatic or non-metastatic status and the remaining 7.8% (*n* = 8) patients reported it wrong as compared to their clinical diagnosis. We analysed which group of patients answered their stage correctly. More patients who had metastatic disease knew the staging correctly (37 of 48 patients) as compared to patients with the non-metastatic disease (35 of 54 patients) (77% versus 64.8% *p* = 0.001). Again, higher level of education in patients did significantly better in knowing the correct stage of the disease.

The patients were also asked about the risk of recurrence/progression. 53.9% (*n* = 55) patients knew that it could recur or progress; 11.7% (*n* = 12) patients reported that it can’t, whereas 34.3% (*n* = 35) patients didn’t have any knowledge about recurrence. None of the studied factors did significantly well in making a difference among patients in knowing that their disease can recur or progress during or after treatment.

## Discussion

This cross-sectional study attempted to assess the knowledge of sarcoma patients about their illness in a tertiary care centre in India. The majority of the patients involved in this study were male with a median age of 31 years. In India, the median age at diagnosis of sarcoma will be between 30 and 40 years and in comparison, in the western population, the age group is 50s. The previous study from India by Bajpai *et al* [15], which analysed the clinical profile of sarcoma patients also showed that the median age of soft tissue sarcoma is about 40 years. The patient cohort in our study involved both university graduates and non-graduates in almost equal numbers. The most common sarcomas in the studied population were Ewing’s sarcoma and synovial sarcoma which is again similar to previous literature from India [15].

The institution, a tertiary care centre has a dedicated sarcoma clinic with an average of 50 outpatient visits every day. Being a high-volume centre, it is required to know the subgroup which needs active intervention apart from baseline education sessions. Thus, it became imperative to analyse the factors that influence the knowledge of patients with sarcoma, especially to identify the vulnerable group which required more active intervention for better understanding.

We found that education and higher socioeconomic status were the only factors that made significant contribution in the patients to understand the disease. Even though we didn’t design a study to prospectively follow these patients, we observed that patients who were educated and in good socioeconomic condition have probably better access to the Internet.

Patients with lower education or illiterate, who belonged to lower socioeconomic status are the worst hit population who requires more attention. These patients should be educated about their illness during each visit and ongoing treatment, its side effects and outcome.

Furthermore, since sarcoma is a rare disease and accounts for 1% of all cancers, lesser data is available in the literature pertaining to the knowledge of the patients regarding the disease [16]. All the previous studies tried to assess the knowledge including patients with common cancer. If the patient is able to know that sarcoma is a rare disease requiring a second opinion in diagnosis and management plan from the centre which is an expert in sarcoma management, then the complete picture of their management and outcome may change in a positive way. In this study, only about half of the patients knew that sarcoma is a rare disease. Since the median time from diagnosis to enrolment in the study is 1 year, patients had sufficient time to reflect upon their disease characteristics.

Even though sarcoma is a rare disease it has multiple subtypes. By knowing the percentage of people who know the specific type of sarcoma they have, we can understand their perspective on the importance of this difficult sarcoma nomenclature in their life. In our study, only about 50% of patients knew the specific type of sarcoma they had which shows the lack of required education among the patients to understand their illness.

Cancer patients should know their disease stage which helps them better participate in discussions with treating doctors about the management plan. It has been reported that patients with advanced cancer are more prone to opt for aggressive end-of-life care when they have inadequate knowledge of their illness [20]. Many of the previous studies [8, 11] had shown that the patient’s understanding of the stage is very poor. Most of them who understood their stage were one having higher education levels, having access to the Internet, female gender and those with a younger age group. In our patient cohort, about two-thirds knew about their disease stage correctly, at least whether the disease is metastatic or non-metastatic. More number of patients with advanced-stage (metastatic) knew about their stage compared to one with an early-stage disease which was statistically significant. This shows that illness understanding is a significant problem in patients with early-stage disease compared to advanced-stage disease. Poor understanding of the early-stage disease was also shown in a previous study by Sivendran *et al* [9]. However, this observation is different from another study by Hinchey *et al* [11] which involved women with non-metastatic breast cancer, where it was demonstrated that patients with early-stage disease had greater illness understanding compared to an advanced stage. The study by Sivendran *et al* [9] was a cross-sectional study conducted by a single centre in North America during the field visit. The study included different type of cancers. Cancer staging involves multiple factors like size of the primary, extension, number of nodes and in some types also grade, and thus may be difficult to remember by the patient. However, metastatic stage is better to understand due to the frequent communication by the treating team related to goals of care and prognosis including the stage of the disease and thus is reported when asked for. Hence, in Sivendran *et al* [9], the higher number of patients with advanced (metastatic) cancer reported their stage correctly. In the latter study we mentioned, Hinchey *et al* [11], the study population involved only stage I to stage III breast cancer. According to this study, the early stage like I and II better understood their cancer. Since there were no metastatic patients involved and it was a single centre study, this observation requires further studies. Furthermore, knowing the stage of their disease also helps patients in understanding the prognosis as reported by systematic meta-analysis [21].

Unfortunately, similar to minimal knowledge about the disease stage, our patients also had poor knowledge with respect to the course of the disease trajectory both in metastatic and non-metastatic settings. Only about half of the patients knew that their disease could progress or recur. This will again impact the seriousness of patients for their illness and hence the compliance of regular follow-up visits.

In many of the previous studies which assessed the knowledge of cancer patients, they found that being a female is a significant factor in a better understanding of their illness [9]. In our study, female patients tend to have better knowledge compared to male counterparts in some of the parameters we assessed. However, this observation was not statistically significant. In India, women have an overall lower education rate than men in India [22]. This may partly explain our observation. In our study, there was no significant effect of these variables on knowledge differences between patients. The questionnaire used in the current study was self-administered to the individual patients and thus the influence of the collective autonomy was minimal.

### Limitations

The study was carried out at a prominent tertiary health care centre, but the possibility of the selection bias cannot be ruled out and it may not be generalisable to the patient population. Also, a self-designed bilingual questionnaire was used to assess the knowledge, which may have excluded some of the other factors affecting the knowledge regarding cancer illness. The study enrolled all patients referred to our clinic during the study period. Some patients were in their initial stage of evaluation and some had already taken treatment from outside the hospital. This inhomogeneity in the patient population is an important confounding factor.

## Conclusion

We found that despite active interventions in terms of the active interactions of the treating physician with the patients, and social support groups, a knowledge gap was still observed in the sarcoma patients with a lack of understanding of the disease’s nature and prognosis. Thus, more interaction between the patient and physician is required, especially for the less educated subgroups, to communicate effectively to the patients about their diagnosis, nature and prognosis. The need of the hour is to design and implement awareness through educational and interventional programmes for both the public and the doctor community in order to increase early diagnosis, referral to expert facilities and thus better therapeutic management.

## Author contributions

Dr Bharath and Dr Vishwas Kumar Anand analysed and interpreted the patient’s data and drafted the manuscript. Dr Sameer Rastogi, Dr Simran Kaur and Dr Nishkarsh Gupta performed the literature review and drafted the manuscript. All authors read and approved the final manuscript.

## Funding

This research did not receive any specific grant from funding agencies in the public, commercial or not-for-profit sectors.

## Availability of data and materials

Not applicable.

## Declarations

### Ethics approval and consent to participate

Ethical clearance was taken from the Institute’s ethical committee (IECPG-192/27.03.2019).

### Consent for publication

Written informed consent was obtained from the patient for publication of this case report and any accompanying images. A copy of the written consent is available for review by the Editor-in-Chief of this journal.

## Conflicts of interest

The authors declare that they have no competing interests.

## Figures and Tables

**Table:** Education of the head of the family

S. No	Occupation of the head	Score
1	Legislators, senior officials & managers	10
2	Professionals	9
3	Technicians and associate professionals	8
4	Clerks	7
5	Skilled workers and shop & market sales workers	6
6	Skilled agricultural & fishery workers	5
7	Craft & related trade workers	4
8	Plant & machine operators and assemblers	3
9	Elementary occupation	2
10	Unemployed	1

**Table:** Occupation of the head of the family

S. No	Education of the head	Scale
1	Profession or honours	7
2	Graduate	6
3	Intermediate or diploma	5
4	High school certificate	4
5	Middle school certificate	3
6	Primary school certificate	2
7	Illiterate	1

**Table:** Total monthly income of the family

S. No	Updated monthly family income in rupees (2018)	Updated monthly family income in rupees (2020)	Updated monthly family income in rupees (2021)	Score
1	≥126,360	≥199,862	≥123,322	12
2	63,182–126,359	99,931–199,861	61,663–123,321	10
3	47,266–63,181	74,755–99,930	46,129–61,662	6
4	31,591–47,265	49,962–74,755	30,831–46,128	4
5	18,953–31,590	29,973–49,961	18,497–30,830	3
6	6,327–18,952	10,002–29,972	6,175–18,496	2
7	≤6,326	≤10,001	≤6,174	1

**Table:** Kuppuswamy’s socioeconomic status scale

S. No	Score	Socioeconomic class
1	26–29	Upper (I)
2	16–25	Upper middle (II)
3	11–15	Lower middle (III)
4	5–10	Upper lower (IV)
5	5	Lower (V)

**Table 1.** Distribution of different sarcoma types and their characteristics in an involved population.

Type of sarcoma - n (%)	Ewing’s – 24 (23.5%) Synovial – 18 (17.6%) Osteosarcoma – 16 (15.6%) Leiomyosarcoma – 9 (8.8%) Liposarcoma – 3 (2.9%) Undifferentiated pleomorphic sarcoma – 7 (6.8%) Others – 25 (24.5%)
Stage – *n* (%)	Metastatic – 48 (47%) Non-metastatic – 54 (53%)
Origin – *n* (%)	Soft tissue sarcoma – 66 (64.7%) Bone sarcoma – 36 (35.3%)

**Table 2.** Differential in the knowledge of sarcoma stage concerning sociodemographic characteristics.

Characteristics	Cancer or not (n, %, odds)	Stage (n, %, odds)	Rarity (n, %, odds)	Recurrence or progression (n, %, odds)
**Age group** ≤ 31 years >31 years	44, 86.2%, 0.83 45, 88.2%, 1.19 P = 0.950	35, 68.6%, 0.87 37, 72.5%, 1.14 P = 0.757	25, 49.0%, 0.67 30, 58.8%, 1.48 P = 0.670	24, 47.0%, 0.57 31, 60.7%, 1.74 P = 0.318
**Gender** Male Female	55, 85.9%, 0.71 34, 89.4%, 1.39 P = 0.725	44, 68.7%, 0.68 29, 76.3%, 1.46 P = 0.175	31, 48.4%, 0.54 24, 63.1%, 1.82 P = 0.054	35, 54.6%, 1.0 20, 58.8%, 0.92 P = 0.90
**Education** Less than 12 years of schooling More than 12 years of schooling	29, 76.3%, 0.21 60, 93.7%, 4.58 P = 0.024	22, 57.8%, 0.38 50, 78.1%, 2.60 P = 0.032	12, 31.5%, 0.22 43, 67.1%, 4.46 P = 0.002	18, 47.3%, 0.65 37, 57.8%, 1.52 P = 0.489
**Socio-economic status** Upper and middle class Lower class	47, 95.9%, 6.15 42, 79.2%, 0.16 P = 0.037	37, 75.5%, 2.90 35, 66%, 0.34 P = 0.569	33, 67.3%, 1.58 22, 41.5%, 0.63 P = 0.022	29, 59%, 1.50 26, 49%, 0.66 P = 0.590

**Table 3.** Demographic details of the patients.

Demographic variables	Value
Age (median ± SD)	31.5 ± 14.37 years
Gender - *n* (%) 1. Male 2. Female	64 (62.7%) 38 (37.3%)
Education - *n* (%) 1. ≤12 years of schooling 2. >12 years of schooling	34 (33.9%) 68 (66.7%)
Socio-economic status – *n* (%) 1. Upper 2. Upper middle 3. Lower middle 4. Upper lower 5. Lower	8 (7.84%) 23 (22.54%) 18 (17.64%) 38 (37.25%) 15 (14.70%)

## Annexure 1

(Questionnaire)

**Understanding of illness in sarcoma patients in an Indian setting**

1. 1.UHID number:

2. Name:

3. Mobile number:

4. Age in years:

5. Gender:

6. Place where you live:

7. Language:

8. Occupation (mark only one)

a. Unemployed

b. Elementary occupation

c. Plant and machine operator and assembler

d. Craft and related workers

e. Skilled agricultural and fishery worker

f. Skilled market and shop sales worker

g. Clerk

h. Technician and associate professional

i. Professional

j. Legislator, senior officer or manager

k. Student

9. Education (mark only one)

a. Illiterate

b. Primary school certificate

c. Middle school certificate

d. High school certificate

e. Intermediate or diploma

f. Graduate

g. Profession or Honours

10. Relationship status

a. Profession or Honours Single

b. Profession or Honours Married

c. Profession or Honours Other

11. Caregiver (relation with you)

a. Spouse

b. Parents

c. Son/Daughter-in-law

d. Daughter/Son-in-law

e. Other

12. Education of caregiver (mark only one)

a. Illiterate

b. Primary school certificate

c. Middle school certificate

d. High school certificate

e. Intermediate or diploma

f. Graduate

g. Profession or Honours

13. Your relation to the head of the family:

14. Education of the head of the family

a. Illiterate

b. Primary school certificate

c. Middle school certificate

d. High school certificate

e. Intermediate or diploma

f. Graduate

g. Profession or Honours

15. Occupation of the head of the family

a Illiterate

b. Primary school certificate

c. Middle school certificate

d. High school certificate

e. Intermediate or diploma

f. Graduate

g. Profession or Honours

16. Monthly family income (mark only one)

a. <or=Rs.6,323 (score 1)

b. Rs.6,327–Rs.18,949 (score 2)

c. Rs.18,953–Rs.31,589 (score 3)

d. Rs.31,591–Rs.47,262 (score 4)

e. Rs.47,266–Rs.63,178 (score 6)

f. >Rs.126,360 (score 12)

17. How many doctors have you seen before coming to AIIMS?

18. Is your illness cancer

a. Yes

b. No

c. I don’t know

19. If your answer is no, then specify your illness

20. Is it sarcoma?

a. Yes

b. No

c. I don’t know

21. If yes, then write the specific type of sarcoma:

22. Is the illness common or rare?

a. Common

b. Rare

c. I don’t know

23. Type of sarcoma you are suffering from/

a. Bone

b. Soft tissue

c. I don’t know

24. Is it metastatic?

a. Yes

b No

c. I don’t know

25. What treatment you have received?

a. Surgery

b. Chemotherapy

c. Radiotherapy

d. None

26. Does this disease recur or progress?

a. Yes

b. No

c. I don’t know

27. What are the factors that lead to this illness?

## Annexure 2

**Modified Kuppuswamy’s socioeconomic status classification**
